# Apolipoproteins as potential communicators play an essential role in the pathogenesis and treatment of early atherosclerosis

**DOI:** 10.7150/ijbs.86475

**Published:** 2023-08-21

**Authors:** Yang Li, Xinyi Luo, Zhenglai Hua, Xiaoxia Xue, Xiangpeng Wang, Mingshi Pang, Tieshan Wang, Aiping Lyu, Yuanyan Liu

**Affiliations:** 1 School of Chinese Materia Medica, Beijing University of Chinese Medicine, Beijing 100029, China.; 2 Beijing Research Institute of Chinese Medicine, Beijing University of Chinese Medicine, Beijing, China.; 3 School of Chinese Medicine, Hong Kong Baptist University, Kowloon, Hong Kong 999077, China.

**Keywords:** Apolipoproteins, Potential communicators of lipoproteins, The pathogenesis of early atherosclerosis, Treatment of early atherosclerosis

## Abstract

Atherosclerosis as the leading cause of the cardiovascular disease is closely related to cholesterol deposition within subendothelial areas of the arteries. Significantly, early atherosclerosis intervention is the critical phase for its reversal. As atherosclerosis progresses, early foam cells formation may evolve into fibrous plaques and atheromatous plaque, ulteriorly rupture of atheromatous plaque increases risks of myocardial infarction and ischemic stroke, resulting in high morbidity and mortality worldwide. Notably, amphiphilic apolipoproteins (Apos) can concomitantly combine with lipids to form soluble lipoproteins that have been demonstrated to associate with atherosclerosis. Apos act as crucial communicators of lipoproteins, which not only can mediate lipids metabolism, but also can involve in pro-atherogenic and anti-atherogenic processes of atherosclerosis via affecting subendothelial retention and aggregation of low-density lipoprotein (LDL), oxidative modification of LDL, foam cells formation and reverse cholesterol transport (RCT) in macrophage cells. Correspondingly, Apos can be used as endogenous and/or exogenous targeting agents to effectively attenuate the development of atherosclerosis. The article reviews the classification, structure, and relationship between Apos and lipids, how Apos serve as communicators of lipoproteins to participate in the pathogenesis progression of early atherosclerosis, as well as how Apos as the meaningful targeting mass is used in early atherosclerosis treatment.

## 1. Introduction

Atherosclerosis is considered to be a chronic inflammatory disease of abnormal accumulation of cholesterol, mainly in the large and medium arteries, leading to high morbidity and mortality worldwide^1^-^3^. Atherosclerosis is a long-term and progressive disease that usually progresses from early fatty streak to fibrous plaque and eventually to highly perilous atheromatous plaque. During this development, the early stage of atherosclerosis is a pivotal period for its progression and regression. Without timely intervention, the atherosclerotic lesions will be further aggravated, and the acute rupture of unstable atheromatous plaque may cause sudden thrombotic occlusion of the artery and fatal clinical complications, including myocardial infarction, stroke, pulmonary embolism and peripheral artery disease^4^-^6^.

Therapeutic strategies for early atherosclerosis mostly revolve around the reduction of lipids levels. The lipids serve as the source of energy and nutrient for the body, mainly including cholesterol, triglycerides (TG) and phospholipids (PL), which are insoluble in water and cannot exist independently in plasma. More importantly, plasma amphiphilic apolipoproteins (Apos) possess the property of interacting with both lipids and water, and act as indispensable carriers of lipids transport. Lipoproteins can be formed by the bind of lipids and Apos, including chylomicron (CM), very low-density lipoprotein (VLDL), intermediate-density lipoprotein (IDL), low-density lipoprotein (LDL) and high-density lipoprotein (HDL), which can transport lipids to various tissues of the body for metabolism and utilization^7^. Along with lipids transport function, Apos also act as communicators of lipoproteins to mediate the metabolism of lipids via the interaction with enzymes and cellular receptors. Recently, massive studies pay more attention to atherogenic LDL and anti-atherogenic HDL, which are associated with cardiovascular disease risk and antagonistic effects in atherosclerotic development^8^,^9^. The atherogenic property of LDL is primarily attributed to its role as a carrier that transports hepatic cholesterol to peripheral tissues, while HDL enhances reverse cholesterol transport (RCT) to accelerate superfluous cholesterol emigration from peripheral tissues to the liver identified as conferring protection against atherosclerosis^8^,^10^. Remarkably, Apos, as functional communicators of lipoproteins, play a crucial role in the interaction between lipoproteins and external objects, so it may participate in the initiation and advance of atherosclerosis.

Accumulating evidences demonstrate that Apos are closely associated with atherosclerotic disease and serve vital regulatory roles in a broad spectrum of key events in early atherosclerosis^11^-^15^. Atherosclerosis commences with the subendothelial retention and aggregation of cholesterol-rich LDL, which is mediated by the interaction of Apos and extracellular matrix (ECM) components such as proteoglycans (PGs)^11^,^12^,^16^. In addition, Apos act as communicators of lipoproteins to irritate oxidative stress and motivate endothelial cells (ECs) and monocytes activation via initiating related signaling pathways. Apos-mediated the activation of ECs may generate multiple cytokines, such as interleukin (IL)-6, IL-8, intercellular adhesion molecule-1 (ICAM-1), vascular cell adhesion molecule-1 (VCAM-1) and so on, which incite the recruitment of monocytes and adhesion to ECs^17^-^19^. Then, monocytes pass through the endothelial tissue and transform into macrophages. ECs and macrophages can be activated by the induction of Apos, secreting inflammatory cytokines to promote the migration and proliferation of smooth muscle cells (SMCs). Trapped LDL within subendothelial space tends to be oxidized and generate a pro-atherogenic form of oxidized LDL (ox-LDL), which can be taken up unrestrictedly in macrophages and SMCs by binding Apos to cell surface receptors, inducing lipid-laden foam cells formation. A large deposition of foam cells within the subendothelial area is a characteristic feature of early atherosclerosis^2^,^20^. Significantly, various cytoprotective Apos, as a defense mechanism of the stress response, generally can be upregulated and accumulated in the lesions and exert manifold conducive effects on the attenuation of atherosclerosis. These conducive effects include the refrain of LDL retention and aggregation, the interference of oxidative stress-related signaling pathways, the inhibiton of ECs and macrophages activation, the modulation of SMCs migration and proliferation and the facilitation of RCT in macrophage cells, which can subsequently reduce foam cells formation and dramatically reverse the progression of atherosclerosis^7^,^21^-^25^.

Apos, as communicators of lipoproteins, play crucial functions in the process of early atherosclerosis. It is worth noting that Apos may be triggered as meaningful targeting mass to ameliorate atherosclerosis via masking the adverse properties of atherogenic Apos and sufficiently amplifying the advantageous properties of anti-atherogenic Apos. Two therapeutic strategies could be taken to synergistically confront the development of early atherosclerosis, one is to regulate endogenous Apos expression and the other is turning to exogenous anti-atherogenic Apos mimetic peptides. In this review, we summarize the classification, structure, and relationship of Apos with lipids, how Apos act as communicators of lipoproteins to affect the development of early atherosclerosis, and even how Apos act as meaningful targeting mass applied for atherosclerosis treatment.

## 2. Apos

Apos are diverse and possess a variety of biological functions. The amphiphilic nature of Apos enables to interact with lipids and water. Since cholesterol, PL, TG and other lipids are insoluble in water and cannot exist alone in plasma, Apos can combine with lipids to form soluble lipoproteins, and the lipids are transported to body tissues in the form of lipoproteins for metabolism and utilization^7^. In addition to as carriers of lipids transport, Apos also serve as communicators of lipoproteins to mediate the interaction between lipoproteins and external objects, which are involved in lipid metabolism in lipoproteins through adjusting enzymes activity and binding to cell surface receptors.

### 2.1 Classification of Apos

Apos are generally classified into five categories, such as A, B, C, D and E, each of which has subcategories, such as AI, AII, AIV and AV in class A, B48 and B100 in class B, CI, CII and CIII in class C, E2, E3 and E4 in class E. So far, more than 20 Apos have been found^26^-^28^. In addition to the above mentioned, classical Apos also contain Apo F, Apo H, Apo J, Apo M and so on. In this review, the Apos mainly refers to Apo A, Apo B, Apo C, Apo E, Apo M and Apo J.

Apos are also divided into exchangeable and non-exchangeable Apos based on their movement and exchange properties between different lipoproteins. Exchangeable Apos can be separated from one lipoprotein and recombined with another lipoprotein during circulation, apparently confering lipoproteins orientation and the ability to interact with cell surface receptors^29^. Exchangeable Apos such as Apo A, Apo C and Apo E must be water-soluble to be exchanged between lipoproteins, while Apo B, as a non-exchangeable apolipoprotein, is highly insoluble in an aqueous medium without interchangeable features with other lipoproteins particles^29^,^30^. Non-exchangeable Apo B retains in a single lipoprotein particle from biosynthesis to catabolism. As well, tracer experiments demonstrated that an Apo B molecule remained together with the parent lipoprotein particle throughout its existence^31^,^32^.

### 2.2 The structure of Apos

**Figure 1:**
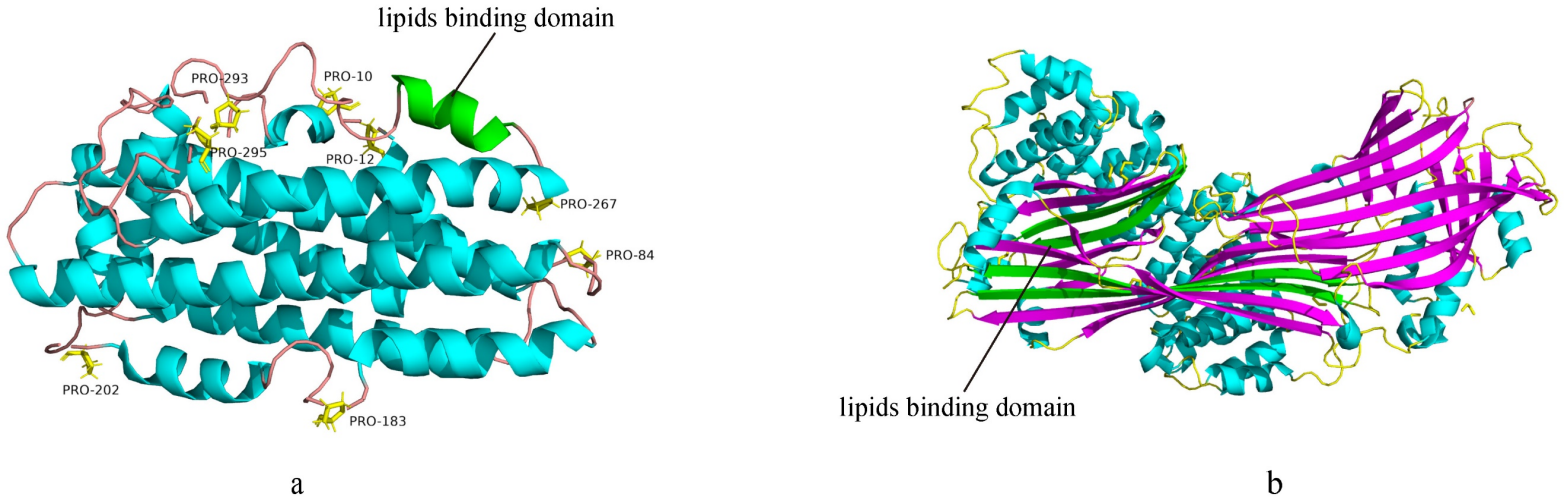
(a) The 3D structure of exchangeable Apos (PDB:2L7B). AαHs are colored blue and green, and the green AαHs represent the lipids binding domain. The proline residues are rendered in stick representation and are colored yellow. The smooth lines represent the backbone of the exchangeable Apos structure and are colored pink. (b) The 3D structure of nonexchangeable Apos (PDB:6I7S). AαHs are colored blue, and AβS are colored purple and green. The green AβS represent the lipids binding domain. The smooth lines represent the backbone of the non-exchangeable Apos structure and are colored yellow.

Exchangeable Apos, sharing unique structure related to their biological functions, are soluble in aqueous solutions and transport between various lipoproteins depending on changes in lipoproteins size and lipids composition^29^. The representative structural character is owning tandem 11-mer or 22-mer domains, usually starting from proline residues and forming amphiphilic α-helices (AαHs). The exchangeability of exchangeable Apos is largely attributable to the content and/or structure of amphiphilic helical motifs^33^. AαHs, the secondary structural motif responsible for the reversible lipids binding, are the universal structural character of exchangeable Apos and are vital structural members for these exchangeable Apos functions (Figure 1a). Apos exchange is indispensable for lipoproteins metabolism, and is considered to generate in the lipid-deficient condition that also serves as major receptors for cellular cholesterol and PL. Besides, the exchangeability of Apos requires dynamic Apos structures to accommodate different lipoproteins surface^34^. Recent findings indicate that the tertiary structures of Apo AI and Apo E are correlated among exchangeable Apos. Apo AI and Apo E are characterized by a series of proline discontinuous AαHs repeats of 11-mer or 22-mer amino acids that exhibit helix-bundle conformation in lipid-free conditions, and their C-terminal domains are primarily associated with lipids binding. Apo C is incapable of constituting helix-bundle conformation attributed to its limited AαHs^35^.

The Apo B, non-exchangeable apolipoprotein, possesses alternative AαHs and amphipathic β-sheet (AβS) domains^32^,^36^ (Figure 1b). The N-terminal of Apo B is homologous to lipovitellin and microsomal triglyceride transfer protein^37^. The AβS domains with a width of approximately 30Å are composed of antiparallel β-sheets, and are essential for lipoprotein assembly and irreversible lipids binding. Apo B48 contains only the β_1_ domain, while Apo B100 contains both β_1_ and β_2_ domains^32^,^38^. Moreover, AαHs domains stabilize electrostatic interactions via expansion and constriction of the PL belt, thus maintaining lipoprotein structural integrity^39^. However, the three-dimensional structure of Apo B remains unclear. Unlike most Apos, Apo M displays a hydrophobic binding pocket similar to lipocalins that enable biological actions. The tertiary structure of Apo M consists of antiparallel β-barrel sheets surrounding the internal ligand binding pocket, flanked by an external α-helix^14^,^40^,^41^.

### 2.3 Relationship between Apos and lipids

Apo are important serum proteins embedded on the surface of lipoproteins particles, endowing lipids with a soluble form. Apos, as communicators of lipoproteins, are also involve in lipids metabolism through modulating enzymes activity and binding to cellular receptors.

Apos combine with lipids in order to form various densities and sizes of lipoproteins that enable to transport hydrophobic mediators in hydrophilic plasma. Lipoproteins are classified into CM, VLDL, IDL, LDL and HDL in ascending order according to hydrated density^42^. In Apo A, Apo AI and Apo AII are mainly present in HDL, and Apo AI exists in CM and VLDL as well^43^,^44^. Physiologically, the majority of circulating Apo AIV is lipid-free or associated with CM, while the remaining proportions are related to HDL^7^,^45^. Apo AV predominately exists in CM, VLDL, and HDL^33^,^46^. In Apo B, Apo B48 is present in CM and Apo B100 exists in VLDL, IDL and LDL^37^. In Apo C, Apo CI, Apo CII and Apo CIII are related to CM, VLDL and HDL. Moreover, Apo CI and Apo CIII are respectively associated with IDL and LDL^47^-^49^. Apo E is secreted as a component of CM, VLDL and HDL^33^. Except for relating to HDL, Apo M is also associated with LDL, VLDL and CM^41^. Apo J also referred to as clusterin acts extracellularly or intracellularly as a chaperone and is mainly carried by HDL, LDL and VLDL^50^,^51^ (Table 1).

Apos act as communicators of lipoproteins to regulate lipid metabolism in lipoproteins by activating or suppressing enzymes. Examples are Apo AI, Apo AIV, Apo AV, Apo CI and Apo E, they may act as activators of lecithin cholesterol acyltransferase (LCAT), which induces free cholesterol (FC) on HDL surface esterified into cholesterol esters (CE) and facilitates CE to get into nascent HDL core to form mature HDL^35^. However, Apo CIII inhibits LCAT activity. Apo CII works as an obligatory activator for lipoprotein lipase (LPL) that promotes TG hydrolysis in triglyceride-rich lipoproteins (TRL). LPL activity is quite low without the presence of Apo CII^48^. Apo CI and Apo CIII are recognized as inhibitors of LPL. They are also shown to inhibit hepatic lipase (HL), while Apo E activates HL^13^,^52^,^53^. Apo AIV and Apo E increase the activity of cholesteryl ester transfer protein (CETP), a pivotal alternative approach for transporting CE to the liver, affecting RCT overall rate^7^,^13^. Nevertheless, Apo CI levels are inversely correlated with CETP *in vitro*^47^.

Apos, as communicators of lipoproteins, may initiate or inhibit lipid uptake from lipoprotein particles by interacting with specific cellular receptors. Apo B as a ligand is essential for LDL binding to LDL receptor (LDLR), facilitating cholesterol ingestion in peripheral tissues and the liver^54^,^55^. Apo AI interacts with hepatocellular surface scavenger receptor class B type I (SR-BI) to trigger CE uptake by hepatocytes for further metabolism^56^. Additionally, as a functional ligand of multiple receptors including LDLR, LDL receptor-related protein (LRP) and heparan sulfate proteoglycans (HSPG), Apo E facilitates the clearance of TRL particles, especially VLDL, VLDL and CM remnant particles, and regulates plasma cholesterol and TG levels 13,57. Besides, Apo E motivates HDL to enter into liver cells for metabolism. In humans, Apo E-containing lipoproteins are cleared faster than those lacking Apo E^58^. Apo C appears to restrain the clearing function of Apo E. Apo CI could impair remnant clearance via LDLR and LRP mediated by Apo E and block the VLDL binding to its receptor^52^. As well, Apo CIII retards TRL remnants' catabolism ascribed to its disturbance of interaction of Apo B or Apo E with hepatic receptors^15^.

## 3. Role of Apos in subendothelial retention and aggregation of LDL

**Figure 2:**
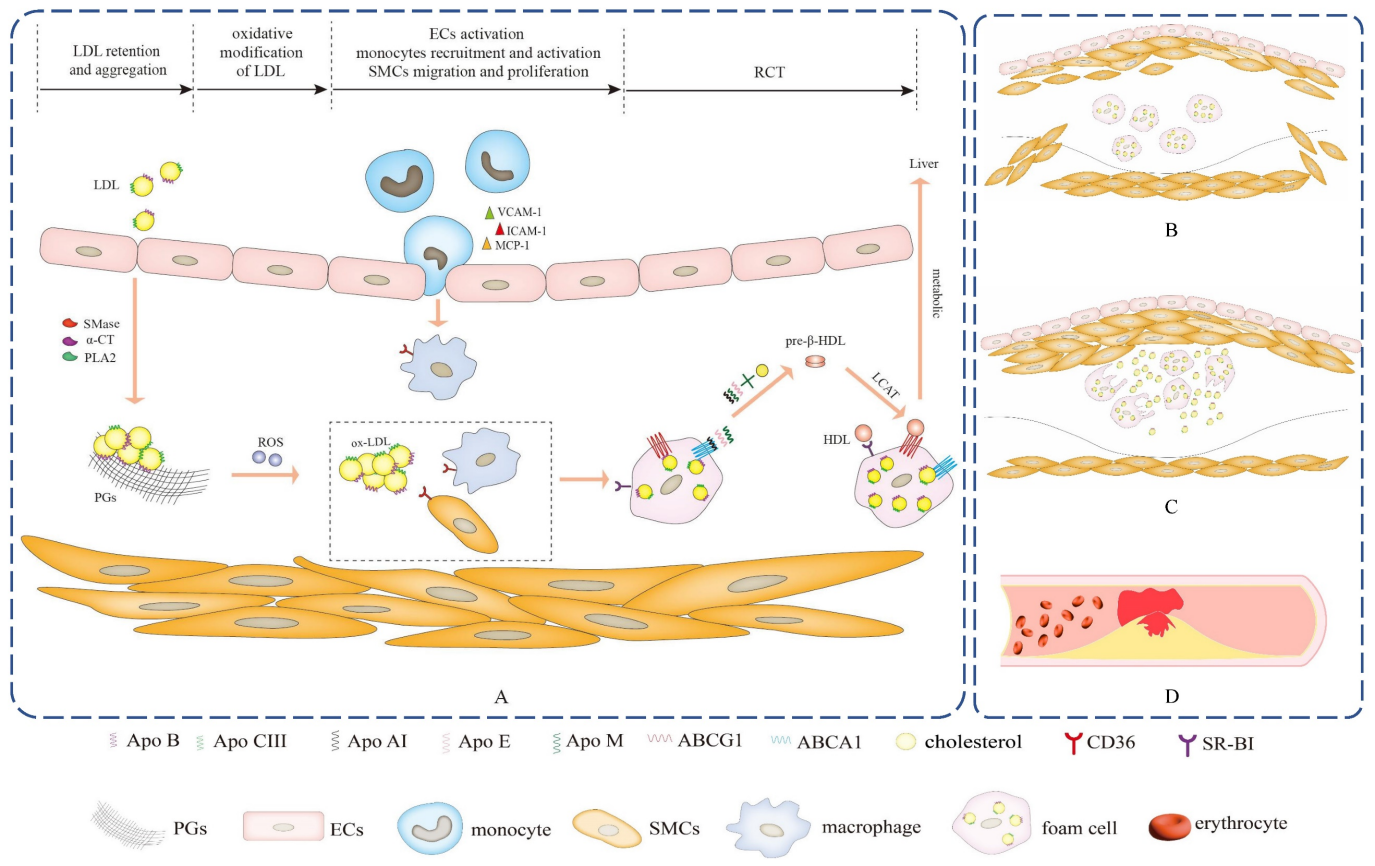
** The progression of atherosclerosis.** (A) Apos serve as communicators of lipoproteins to participate in the pathogenesis of fatty streak, which is the early stage of atherosclerosis and is reversible. (B) As the lesion progresses, a large number of SMCs migrate into the intima, and SMCs proliferate and secrete a large amount of extracellular matrix to significantly thicken the intima of the lesion, which is the fibrous plaque stage. (C) The lesion progresses further, foam cells die and disintegrate, releasing their contents, which is the atheromatous plaque stage. (D) Unstable atherosclerotic plaques are prone to rupture and can cause sudden thrombotic occlusion of the artery, blocking blood flow and resulting in fatal clinical complications, including myocardial infarction, stroke, pulmonary embolism and peripheral artery disease.

Apo B acts as a major communicator of LDL can spark subendothelial retention of excessive LDL by interacting with ECM components PGs, which is an initial step of atherosclerosis^11^,^12^. Other Apos compositions in LDL, as well as phospholipases and proteases-triggered LDL modification may cause conformational changes in Apo B, which increase the binding of Apo B to PGs and lead to aggregation into large macromolecular complexes, further aggravating the retention of LDL (Figure 2A). As a stress response, several Apos protect against lipoproteins aggregation, exerting anti-atherogenic effects and inhibiting atherosclerotic development.

### 3.1 Apos trigger subendothelial retention of LDL

The endothelial layer maintains integrity upon the majority of atherosclerotic progression, whereas it is highly permeable to LDL and the exact molecular mechanism promoting this increase in permeability is unclear. Other than LDL particles, the small Apo B-containing lipoproteins with sizes about 70 nm, such as CM remnants, VLDL and IDL, could effectively pass through ECs^59^. Experimental researches indicate that PGs versican, biglycan and perlecan are ECM components within the subendothelial region and are believed to show a significant role in the process of lipoproteins retention and aggregation^59^. PGs are macromolecules made up of core proteins and long-side chain glycosaminoglycans (GAGs), composed of duplicated disaccharide units owning negatively charged groups^37^. The positively charged amino acids in Apo B could bind to PGs via negatively charged GAGs^37^,^60^. Eight binding sites in Apo B100 are identified to interact with PGs. Site B (residues 3359-3369) serves as a functional combining site in Apo B100 and others without function possibly bury in the LDL surface lipid layer^61^. Similar to LDL, smaller particles like CM remnants, VLDL and IDL are easily trapped and accumulated via the electrostatic interactions^62^. Furthermore, other Apos compositions of LDL affect their combination with PGs. Apo E can mediate the binding of lipoproteins to negatively charged GAGs chains of arterial PGs and the co-localization of Apo E and biglycan in lesions has been testified^16^. As well, exchangeable Apo CIII content in LDL is related to enhanced affinity with PGs biglycan^16^,^61^,^63^. Unlike Apo B and Apo E, Apo CIII cannot directly combine with PGs due to a lack of positively charged amino acid^53^. The enhanced combination may be attributed to conformational changes connected with high Apo CIII content, making Apo B or Apo E more approachable to PGs^64^,^65^. Moreover, a high Apo CIII/Apo B ratio reduces the lipids composition content of LDL and these changes may contribute to increased membrane fluidity and enable Apo B to form a more conducive conformation for PGs binding^53^,^63^.

### 3.2 Conformational changes in Apos induced by enzymes promote the aggregation of LDL

Following subendothelial retention, lipoproteins are more easily modified by phospholipases and proteases, including sphingomyelinases (SMase), phospholipaseA2 (PLA2) and α-chymotrypsin (α-CT). These enzymes promote the formation of hydrophobic domains in Apo B of lipoproteins, facilitating aggregation and fusion of retained lipoproteins^21^,^64^, which form larger lipoprotein-like particles and are hard to leave the arterial walls. As the conformation of Apo B100 on the LDL surface may be related to lipids composition and content as well as LDL particle size, thus other combinative sites could be exposed and play a role in modified LDL^16^. Site A (residues 3148-3158) in Apo B100 plays a role in PLA2-modified LDL and acts synergistic effect with site B to strengthen the bond to PGs^37^. The changes in LDL surface lipid monolayer induced by SMase result in significant changes in the conformation of Apo B100, manifesting as decreased α-helical and increased β-sheet content. SMase-induced conformational changes appear to cause exposure of specific hidden regions of Apo B100 and increase surface hydrophobicity of LDL particles that are crucial for aggregation^66^. α-CT induces Apo B proteolysis and proteolytic fragments released from LDL to stimulate the fusion of LDL^21^. Apo CIII acts as a SMase activator, which makes Apo CIII enriching in LDL more susceptible to hydrolysis and aggregation by SMase^67^.

### 3.3 Apos protect against the aggregation of LDL

Although some Apos accelerate atherosclerotic progression via promoting lipoproteins accumulation within the subendothelial space, several Apos exhibit inhibition of lipoproteins aggregation and anti-atherogenic effects. Apo AI could potently bind LDL surface lipids, thereby interfering with surface lipid distribution and stabilizing the conformation of Apo B100, which interrupts aggregation induced by SMase^22^. Besides, Apo J may act controlling effect on aggregation. Apo J concentrations do not increase in normal arteries but in the form of LDL binding in atherosclerotic lesions. Apo J-depleted LDL experiments prove that Apo J presents a positive effect on LDL aggregation. In addition to retarding aggregation, Apo J also makes Apo B less sensitive to proteolysis, demonstrating Apo J as a universal mechanism against LDL aggregation^21^. The mechanism may be associated with the nature of Apo J binding to exposed hydrophobic domains during protein unfolding^68^, thus preventing the interaction hydrophobic surfaces-mediated interactions and subsequent aggregation of LDL particles^67^,^69^.

## 4. Role of Apos in oxidative modification of subendothelial LDL

**Figure 3:**
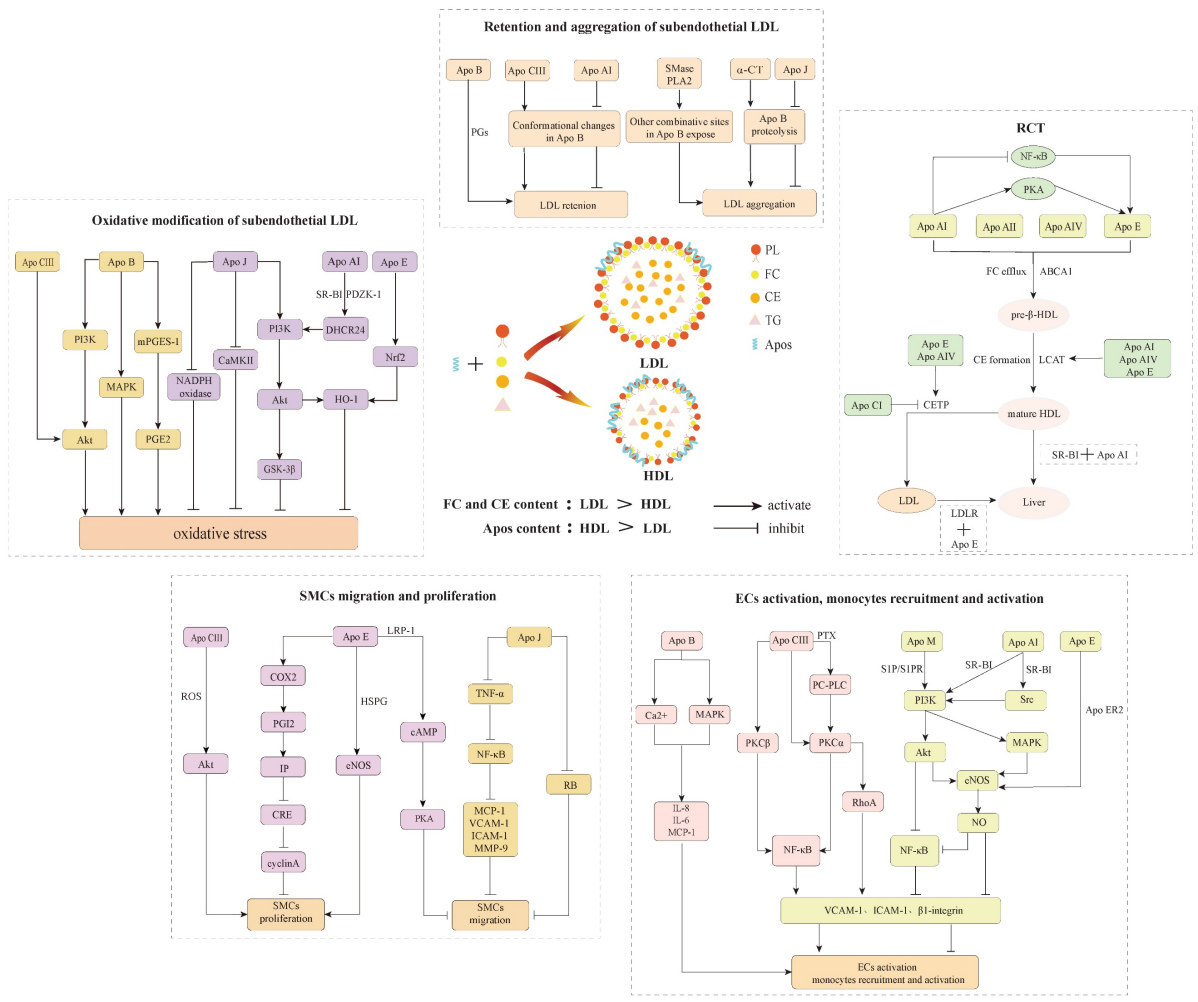
Apos participated processes of retention and aggregation of subendothelial LDL, oxidative modification of subendothelial LDL, ECs activation, monocytes recruitment and activation, SMCs migration and proliferation, as well as RCT.

LDL-associated Apos act as communicators on oxidative stress-related signaling pathways to instigate reactive oxygen species (ROS) production and lead to the generation of atherogenic ox-LDL that has been widely identified as a major determinant driving the pathogenesis of atherosclerosis. Indeed, ox-LDL and its bioactive oxidized byproducts, such as lipid peroxides, aldehydes, hydroxyl sterols, and lysophosphatidylcholine, exhibit pro-atherogenic properties and contribute to atherogenesis by affecting manifold biological processes, containing the induction of cholesterol accumulation in macrophages as well as potent proinflammatory, proapoptotic and cytotoxic activities^70^,^71^. Meanwhile, various Apos accumulate in early atherosclerotic lesions and are considered as a defense mechanism against oxidative stress, which exerts antioxidative effects directly and indirectly through two synergistic manners to restrain atherosclerotic development *in vivo*. One is involved in oxidative stress-associated signaling pathways that directly remove free radicals and prevent LDL oxidation, and the other interacts with oxidant and LDL oxidation byproducts and blocks their harm in atherosclerosis (Figure 3).

### 4.1 Apos promote oxidative stress-related signaling pathways

On most occasions, LDL circulating in plasma is protected from oxidation with the presence of antioxidants^72^. It is currently acknowledged that oxidative modification of LDL occurs locally within the subendothelial space. The prolonged retention of LDL is mainly through Apo B as a communicator to trigger oxidative stress and induce ox-LDL formation. Apo B is a potent stimulator of the microsomal prostaglandin E synthase-1 (mPGES-1) pathway, contributing to the augmentation of prostaglandin E2 (PGE2) biosynthesis in early atherosclerotic lesions, which enhances vascular oxidative stress to a certain extent^17^. Apo B also stimulates platelet to interact with leukocytes in a p-selectin-dependent manner through activation of phosphatidylinositol 3-kinase (PI3k)/Akt and mitogen-activated protein kinase (MAPK), increasing leukocyte activation and ROS generation^73^. Furthermore, the effect of Apo CIII in LDL on ROS production was positive, and elevated ROS and lipid peroxides induced by Apo CIII were detected in aortic lesions in Apo CIII transgenic mice, which may be related to Akt phosphorylation^18^. The oxidative modification of Apo B mediates recognition of ox-LDL by scavenger receptors, facilitating unrestricted cholesterol uptake and accumulation in macrophages^74^.

### 4.2 Apos restrain oxidative stress-related signaling pathways

To maintain the prooxidant-antioxidant balance, several Apos also act as communicators of lipoproteins to synergistically attenuate oxidative damage by inhibiting oxidative stress-related signaling pathways. Apo J is enhanced in atherosclerosis as a protein sensor of oxidative stress, which significantly inhibits ROS production stimulated by ox-LDL through lowering expression related to nicotinamide adenine dinucleotide phosphate (NADPH) oxidase^75^,^76^. Apo J against detrimental effects of oxidants appears to be dependent on the phosphorylation of Akt/glycogen synthase kinase-3β (GSK-3β) and PI3K to reduce cellular ROS levels^77^,^78^. As well, Apo J exerts a cytoprotective effect against apoptosis caused by ox-LDL. Ma et al.^76^ found that Apo J conferred cytoprotection against ox-LDL cytotoxicity via interfering with ROS/calmodulin-dependent protein kinase II (CaMKII) pathways. Additionally, the antioxidative properties of Apos are also reflected in the activation of the nuclear factor erythrocyte 2‐related factor 2 (Nrf2) pathway. Nrf2 serves a positive role in regulating gene expression in antioxidative stress protection mechanisms^79^. Apo E may act as an antioxidative agent in part by interacting with Nrf2, which promotes the activation of downstream heme oxygenase‐1 (HO‐1)^23^. It has been indicated that HO-1 dramatically lessens atherosclerotic development in mice. Besides, Apo AI increases 3β-hydroxysteroid-Δ24 reductase (DHCR24) level by interacting with SR-BI and PDZ-domain-containing protein 1 (PDZK1), provoking phosphorylation of PI3K/Akt to trigger HO-1 expression to inhibit oxidative stress^80^.

### 4.3 Apos interact with oxidant and LDL oxidation byproducts

Except for inhibiting oxidative stress-related signaling pathways mediated by Apos, other antioxidative properties of Apos that interact with oxidant and LDL oxidation byproducts are well established. Apo AI may act directly as an antioxidant owning to contain multiple methionine groups and it suppresses the formation of lipid hydrogen peroxide, oxidized CE and PL, which lowers cytokines levels such as MCP-1 and macrophage colony-stimulating factor, as well as inhibits monocytes adhesion to ECs mediated by oxidative lipids, further blocking deleterious effects of them in atherosclerosis process^81^,^82^. Ox-LDL induces monocytes to infiltrate subendothelial and transform into macrophages that secrete myeloperoxidase to catalyze ROS intermediates formation and further aggravate LDL oxidation. Apo AIV could lower macrophage-triggered lipoproteins oxidation and copper ion-induced LDL oxidation is inhibited in a dose-dependent manner by rat Apo AIV. Moreover, Apo AIV accommodates intracellular glutathione redox balance and alleviates apoptosis mediated by oxidant^7^. Lipoproteins in Apo E-lacking mice are more prone to be oxidized *in vitro* than in wild-type mice. Evidence of amplified oxidative stress and specific epitopes of LDL oxidation in aortic lesions has been found in Apo E-deficient mice. Although the mechanism is acquainted imperfectly, the antioxidant properties of Apo E are ascribed to bind to copper and iron ions, which may isolate them and thus restrain their involvement in the oxidation process^13^. Furthermore, the disulfide bond of Apo J may decrease ROS content through its cysteine sulfhydryl groups^83^. Except for protecting LDL from copper ion-induced oxidation, Apo M may also enable to binding of oxidized phospholipids through its lipocalin structure, making it an effective inhibitor of LDL oxidation^14^,^84^. As well, Apo AI, Apo AII and Apo AIV may facilitate the removal of oxidized PL from LDL to attenuate their harmful effects^71^.

## 5. Role of Apos in the foam cells formation

Several Apos as communicators of lipoproteins are involved in inflammatory-related signaling pathways to incite robust proinflammatory activity, contributing to the occurrence of several key events involved in foam cells formation, such as ECs activation, monocytes recruitment and activation, as well as SMCs migration and proliferation. Macrophages are mainly derived from activated monocytes and SMCs that migrate from arterial media to intima. Subsequently, macrophages can avidly engulf ox-LDL by the Apos binding to cellular receptors, leading to the formation of lipid-rich foam cells that are typical hallmarks of early atherosclerosis.

### 5.1 Role of Apos in ECs activation, monocytes recruitment and activation

Apos serve as communicators of lipoproteins to participate in multiple inflammatory responses through motivating ECs-monocytes interactions and exert specific effects on atherogenesis. Research has verified that Apo CIII alone or as a lipoproteins component induced ECs activation and monocytes adhesion *in vitro*^15^,^19^. VLDL and LDL with Apo CIII expedited monocytes adhesion to ECs compared with those without Apo CIII 85,86. Ying et al.^87^ fed Apo CIII transgenic/LDLR null and LDLR null littermates with an atherogenic diet, and they found that Apo CIII transgenic mice increased aortic inflammation accompanied by enhanced macrophages infiltration and VCAM-1 expression. Under static or flowing conditions, Apo CIII promotes monocytes adhesion to ECs via protein kinase C alpha (PKCα) and RhoA-mediated beta1 (β1)-integrin activation. Besides, ECs-monocytes interactions irritated by Apo CIII may relate to pertussis toxin (PTX)-sensitive G protein and phosphatidylcholine-specific phospholipase C (PC-PLC) that participates in PKCα activation and further activates nuclear factor-kappaB (NF-κB) and upregulates β1-integrin expression in monocytes^88^. As well, Apo CIII facilitates adhesion molecules expression in ECs such as VCAM-1 and ICAM-1 in the manner of PKCβ and NF-κB activation, causing atherogenesis via ECs activation and monocytes recruitment^19^. More importantly, Apo B was indicated to activate monocytes and macrophages in *ex vivo* cultured carotid plaques through evoking MAPK and Ca^2+^-dependent signaling pathways, which incites the release of multiple cytokines containing IL-6, IL-8 and MCP-1 involved in monocytes recruitment in atherosclerotic lesions^17^.

The aforesaid findings reveal that some Apos as dangerous signals participate in foam cell formation. Meanwhile, cytoprotective Apos as communicators of lipoproteins may adjust manifold associated signal transduction pathways and actuate resistance to cellular atherogenic response (Figure 3). As a multifunctional protein, Apo E mainly synthesizes in the liver and macrophages, and may function directly in atherosclerotic lesions. Macrophage-derived Apo E shows local cytokine and hormone-like effects on perivascular cells, exerting its atheroprotective effect. For instance, Apo E initiates a signal cascade via interacting with Apo E receptor 2 (Apo ER2), leading to endothelial nitric oxide synthase (eNOS) activation and inducing nitric oxide (NO) generation, and again refraining the expression of VCAM-1 on ECs^24^,^89^. Apo E also restrains the T lymphocytes activation and proliferation^13^. Apo M as a chaperone of sphingosine-1-phosphate (S1P) contributes to the formation of Apo M/S1P complex in HDL, which interacts with S1P receptors (S1PR) to promote vascular barrier function and exert anti‐inflammatory functions^90^,^91^. S1PR1, S1PR2 and S1PR3 exist in vascular tissues, as well as S1PR1 is abundant in ECs^40^,^90^. The interactions between Apo M/S1P complex and S1PR1 are to maintain tight junctions between ECs and intact endothelial barrier^14^. Compared with wild-type mice, vascular permeability in lung tissue is enhanced in Apo M-absent mice, demonstrating that Apo M/S1P is necessary for retaining vascular integrity^92^,^93^. Apo M/S1P prominently reduces inflammatory and adhesion molecules expression. Zheng et al.^94^ suggested that Apo M/S1P mediated PI3K/Akt phosphorylation by S1PR2, inhibiting NF-κB translocation and reducing ox-LDL-induced cytokines secretion in ECs, such as VCAM-1, ICAM-1, tumor necrosis factor-α (TNF-α) and IL-1β. Also, Apo M/S1P may activate PI3K/Akt pathway by S1PR1 and S1PR3 to irritate eNOS expression and NO generation, lessening monocytes adhesion to ECs^95^,^96^. NO acts as a crucial atheroprotective cytokine regulating leukocyte adherence to ECs. Transgenic mice overexpressing Apo AI refrained aortic fatty streak development, ascribing to activation of tyrosine kinase (Src) and PI3K mediated by Apo AI/SR-BI, which results in parallel activation of Akt and MAPK, as well as triggers eNOS expression and NO generation^97^. S1P/S1PR2 increases inflammatory and adhesive molecules such as MCP-1, ICAM-1, VCAM-1, TNF-α and IL-1β through activating NF-κB in ECs. SR-BI and S1PR are co-located in ECs and Apo AI/SR-BI may negatively modulate inflammation incited by S1P/S1PR2 through PI3K/Akt/eNOS/NO/NF-κB signal transduction pathway, decreasing the release of inflammatory and adhesive mediators^98^. Ox-LDL has been reported to activate classical complement cascade to cause leukocyte infiltration and inflammatory response^99^. Complement complexes can be discovered in atherosclerotic lesions, and ECs exposed to complement are activated and improves the production of IL-6, IL-8 and MCP-1, thereby triggering the adhesion of monocytes to ECs^89^. Apo J could interact with complement components to prevent ECs activation through complement cascade and exert antiatherosclerotic effect^100^,^101^.

### 5.2 Role of Apos in SMCs migration and proliferation

Excessive migration and proliferation of SMCs from arterial media to intima are also one of the critical factors promoting atherosclerotic foam cells formation. Inflammatory cytokines derived from Apos-mediated activation of ECs and monocytes stimulate SMCs migration and proliferation. SMCs express various cytokines containing fractalkine, ICAM-1, MCP-1, VCAM-1 and matrix metalloproteinase-9 (MMP-9) under pathological conditions^102^,^103^, further exacerbating ECs activation, monocytes recruitment, SMCs migration and proliferation, which amplifies pro-inflammatory cascades and form a vicious circle. Apos as communicators of lipoproteins act vital effects in SMCs migration and proliferation by modulating related signaling pathways (Figure 3). Apo CIII may stimulate the proliferation of SMCs through Akt phosphorylation mediated by ROS^18^. Apo J partially represses the TNF-α/NF-κB signaling pathway and downregulates MMP-9, adhesion mediators and chemokines expression, blocking the migration of SMCs. Except for taking effect on SMCs migration, Apo J also inhibits SMCs proliferation through reducing retinoblastoma protein (RB) phosphorylation to mediate G1 phase cell cycle arrest^102^. In addition, it has been confirmed that Apo E refrains SMCs migration and proliferation triggered by ox-LDL, serum and platelet-derived growth factor (PDGF) via distinct mechanisms^13^. Apo E suppresses SMCs proliferation after vascular injury *in vivo* and its anti-proliferative effects are partly attributed to the inhibition of the cell cycle^104^. Apo E may trigger cyclooxygenase-2 (COX-2) generation, and promote the synthesis of prostacyclin (PGI2) and the activation of prostacyclin receptor IP, suppressing cyclin A gene expression in cyclic adenosine monophosphate (cAMP)-response element (CRE)-dependent manner and interrupting cycle progression of aortic SMCs^105^. Simultaneously, other anti-proliferative effects mediated by Apo E involve its binding to HSPG and subsequent induction of NOS activition^106^,^107^. Compared to proliferation, the suppression of PDGF-induced SMCs migration by Apo E may be mediated through LRP-1, and their binding stimulates the activation of the cAMP/PKA signaling pathway^13^,^107^.

## 6. Role of Apos in RCT in macrophages or foam cells

It is well established that the continuous influx of cholesterol ultimately exceeds the metabolic capacity of macrophages and thus induces foam cells formation. RCT is responsible for motivating excessive cholesterol efflux from extrahepatic tissues and returns to the liver for further metabolism and excretion^56^,^108^. Pieces of evidence support that the anti-atherosclerotic action of HDL is conventionally ascribed to its participation in RCT^108^,^109^,^110^. More importantly, Apos as significant communicators of lipoproteins are involved in RCT and exert specific effects, which not only serve as cholesterol receptors in extrahepatic cells to motivate new HDL particles generation, but also as cholesterol transport carriers to the liver for metabolism. This suggests that Apos-mediated RCT is the prime mechanism of restraining foam cells formation and facilitating atherosclerotic regression (Figure 2A).

### 6.1 Apos acting as cholesterol receptors facilitate cholesterol efflux from macrophages or foam cells

The initial step of RCT is to mobilize cholesterol efflux from macrophages or foam cells. Lipid-poor Apo AI is the dominant acceptor of cellular FC and PL in an adenosine triphosphate-binding cassette transporter A1 (ABCA1) dependent manner to form nascent discoidal pre-β-HDL that serves as a more active FC acceptor^111^,^112^. Other exchangeable Apos including Apo C and Apo E share the amphipathic helical structure and may also function in this regard^113^,^114^. Indeed, Apo E owns the competence to promote HDL synthesis by combining with ABCA1 independently of Apo AI. It has been demonstrated that Apo E expression mediated by adenoviral motivates discoidal HDL secretion in mice lacking Apo AI^25^. Furthermore, human Apo AI is known to be an effective inducer of endogenous Apo E production from lipid-loaded macrophages and independent of ABCA1-mediated cholesterol efflux, relating to NF-κB inhibition and/or PKA pathway activation^115^. The cholesterol efflux capacity of Apo AII is as efficient as Apo AI and they exert synergistic effects in HDL particles for cholesterol efflux^44^. Evidence reveals that Apo AIV expedites cholesterol efflux and HDL particles in Apo AIV transgenic mice display a higher capacity to lessen intracellular cholesterol content compared with wild-type mice^7^. HeLa cells overexpressing human ABCA1 enhanced cholesterol efflux via binding to Apo AIV, implying that Apo AIV was involved in ABCA1-mediated cholesterol efflux^116^. Besides, Apo AIV was indicated to enhance cholesterol emigration in PL-containing liposomes, comparable to that of Apo AI and Apo E under physiologically relevant concentrations^7^. Apo CI, Apo CII, and Apo M also serve as cholesterol acceptors to potentially inhibit foam cells formation^40^,^52^,^117^. Whereas Apo CI generation by macrophages does not exert a protective effect in atherosclerotic development despite reducing cholesterol accumulation in macrophages^52^.

### 6.2 Apos serving as LCAT activator promote mature HDL formation

In the second step, LCAT catalyzes CE formation on the pre-β-HDL surface via transferring fatty acids from lecithin to FC. Since CE is less soluble than TG, it cannot circulate in exposure to a water environment, thereby it migrates from lipoprotein particles surface to hydrophobic core, rendering discoidal pre-β-HDL into mature spherical HDL^118^. Mature HDL may further mediate FC efflux via other pathways, containing ABCG1 and SR-BI^7^. Apo AI is not the only potent activator of LCAT, which could also be activated by Apo AIV, Apo C and Apo E^111^. In comparison with other Apos, Apo E promotes the enlargement of HDL size ascribed to its interaction with PL polar head groups, contributing to CE enrichment in hydrophobic core^119^.

### 6.3 Apos as cellular ligand mediate cholesterol metabolism in the liver

In the final step, HDL is transported to the liver for metabolism. CE on HDL is mainly eliminated through hepatic surface receptor SR-BI^112^. Apo AI as a ligand interacting with SR-BI mediates CE uptake in HDL, and deficiency of Apo AI induces a reduction in CE selective uptake^56^,^120^. Except for Apo AI, Apo E also mediates HDL ingestion into hepatocytes and other cells as a ligand^58^. While the clearance of HDL may be interfered with Apo CIII. Morton et al.^58^ emphasized that Apo CIII and Apo E functioned antagonistically on HDL metabolism. As a high-affinity ligand, Apo E mediates whole HDL uptake by hepatocytes and the residence time in the circulation is much shorter than that without Apo E. Whereas Apo CIII coexisting with Apo E on HDL inhibits Apo E binding to receptors and abrogates the benefits of Apo E on HDL clearance. Alternatively, CE from HDL could be moved to Apo B-containing lipoproteins through CETP, subsequently delivered to the liver and taken up by LDLR^121^. CETP as a lipid transfer protein participates in lipids exchange and transport among various lipoproteins, which is the substitute way for CE delivery and thus influences the overall rate of RCT^111^. Apo AIV and Apo E increase human plasma CETP activity^7^,^33^. Nevertheless, Apo CI is negatively correlated with CETP *in vitro*^37^.

## 7. Apos as meaningful targeting mass for treatment of early atherosclerosis

Apos as communicators of lipoproteins play important roles in the pathogenesis of early atherosclerotic development. Correspondingly, they may serve as meaningful targeting mass to reverse atherosclerosis via covering up their unfavorable features and fully augmenting their beneficial features. There are two main available strategies triggering Apos for the treatment of early atherosclerosis. One reasonable approach is to regulate the expression of endogenous Apos to inhibit atherosclerosis via reducing the level of endogenous atherogenic Apos or mutating them and urging the synthesis of endogenous anti-atherogenic Apos. When the generation of endogenous Apos is insufficient or difficult to reverse the progression of atherosclerosis, the application of exogenous Apos mimetic peptides exhibiting similar biological features to native Apos is considered to be another supplementary and desirable approach for atherosclerotic therapeutic option.

### 7.1 Endogenous Apos as potential targets for the treatment of early atherosclerosis

Considering that Apos are bound up with the occurrence and progression of early atherosclerosis due to their atherogenic and anti-atherogenic properties, corresponding interventions such as reducing or mutating endogenous atherogenic Apos, as well as elevating endogenous anti-atherogenic Apos levels could retard atherosclerotic development. Remarkably, anti-Apos antisense oligonucleotide (ASO), aiming at Apos mRNA and reducing plasma Apos levels, offers an intriguing therapy for cardiovascular protection. An anti-Apo B ASO effectively lessens hepatic Apo B synthesis and lowers plasma cholesterol, achieving a basic elimination of plasma LDL in LDLR-deficient mice with atherosclerotic lesions. LDL concentrations declined rapidly within 3 days of administration. After 7 days of ASO treatment, the reduction of plasma Apo B concentrations lowered aortic LDL permeability, possibly further reducing LDL retention and aggregation within subendothelial space, which is prior to any detectable changes in foam cells content and size of aortic atherosclerotic plaques^122^. As well, mutating Apos refrains atherosclerotic development through lessening LDL retention. Skålén et al.^123^ created heterogeneous transgenic mice expressing recombinant LDL with a mutation in Apo B100, containing tyrosine replaced tryptophan-4369, basic amino acids in Apo B100 binding site (residues 3359-3369) changed to neutral amino acids, lysine 3363 in Apo B100 converted to glutamic acid and 6-GAGs binding site mutation in Apo B100. The results indicated that recombinant LDL with a mutation in Apo B was retained less than in wild-type mice, owning greatly decreased atherogenic potential. Volanesorsen, a second-generation anti-Apo CIII ASO, has been indicated to selectively lower Apo CIII biosynthesis in whole preclinical tested non-human primates and rodents. Additionally, this drug is tested in healthy volunteers in the primary stage of clinical use, displaying a dose-dependent reduction in Apo CIII levels and being well tolerated^124^,^125^. A specific antibody against Apo CIII is capable of blocking detrimental pro-atherogenic effects, decreasing the expression of ICAM-1 and VCAM-1 in ECs through inactivating PKCβ and NF-κB, and inhibiting monocyte recruitment^49^,^124^. Meanwhile, statin administration effectively inhibits Apo CIII-induced ECs activation and monocyte infiltration by restraining the NF-κB pathway *in vivo*^126^.

An upregulator 4010B-30 has been reported to regulate Apo AI gene expression partly through the peroxisome proliferators-activated receptor γ-mediated pathway, contributing to increase cholesterol efflux in macrophage cells and reduce atherosclerosis^127^. The increase of hepatic Apo AI synthesis by myriocin is associated with the inhibition of extracellular-signal-related kinase phosphorylation^128^. The anti-atherogenic mechanism of myriocin may be linked to promote cholesterol efflux via this pathway^129^. RVX-208, composite molecular belonging to the quinazoline family, acts through an epigenetic mechanism by inhibiting the bromodomain and extraterminal protein to upregulate Apo AI, resulting in the stimulation of RCT^130^. As well, the RVX-208 induced Apo AI messenger ribonucleic acid and protein synthesis in HepG2 cells^131^. African green monkeys treated with RVX-208 for 63 days elevated plasma Apo AI and HDL cholesterol levels up to 60% and 97%, resulting in increased cholesterol efflux via ABCA1, ABCG1 and SR-BI pathway^131^. In addition, the increase in Apo AI concentrations could be indirectly realized by modulating its catabolism. Niacin may raise Apo AI levels primarily by preventing the liver from eliminating Apo AI and augmenting RCT^132^. Elevated circulating endogenous Apo E levels could prevent atherosclerosis in Apo E-absent mice through hepatic gene transfer and Apo E transgenic expression mediated by recombinant adenovirus^13^. Oleic acid promotes Apo E synthesis and secretion in macrophages originating from monocyte at a locus involving post-translational glycosylation^133^. Yang et al.^134^ proved that Apo M was the target for the regulation of lipids by simvastatin. Simvastatin dramatically induces Apo M expression *in vitro* and *in vivo*, and its mechanism is related to inhibiting LXRα and expediting hepatocyte nuclear factor-1α. The regulation of Apo M may be a new way for statins to enhance HDL level and facilitate RCT to prevent atherosclerosis (Table 2).

### 7.2 Exogenous Apos mimetic peptides as supplementary targets for the treatment of early atherosclerosis

Apos mimetic peptides derived straightly from the sequence of Apos or amino acid sequence mimicking their AαHs conformation show pleiotropic anti-atherogenic properties, such as inhibiting LDL retention and aggregation, promoting RCT, as well as antioxidative and anti-inflammatory functions, rendering them to be novel and better exogenous agent potential exogenous agent for preventative and therapeutic atherosclerosis owing to the perspective of clinical applications^135^. Administration of Ac-hE18A-NH2, an Apo E mimetic peptide, to western diet-fed Apo E null mice results in increasing PON-1 activity related to HDL and decreases plasma ROS and lipid hydroperoxide levels^136^. Ac-hE18A-NH_2_ restrains the expression of IL-6, VCAM-1 and MCP-1 in lipopolysaccharide-induced ECs and macrophages, and decreases the adhesion of monocytes to ECs. It is also linked to the generation of pre-β-HDL that may be involved in triggering endogenous Apo E secretion in macrophages and motivating cholesterol efflux in an ABCA1-independent manner, thereby inhibiting foam cells formation^137^. Other Apo E-derived peptides such as ATI-5261 and CS-6253 prompt RCT with analogous efficiency to native Apo E. Human plasma added to ATI-5261 or CS-6253 enhances cholesterol emigration in an ABCA1-dependent way and causes pre-β-HDL formation, as well as mature HDL-ATI-5261 and HDL-CS-6253 motivate SR-BI-mediated hepatic cholesterol uptake. Whereas, ATI-5261 generates muscle toxicity in wild-type mice compared to CS-6253^136^. Ep1.B, another peptide of Apo E, could inhibit early plaque formation in Apo E null mice, effectively replacing Apo E roles^138^. The inhibitory effect of Ep1.B was associated with the reduction of inflammatory monocytes infiltration. Besides, Ep1.B induced monocytes to differentiate into dendritic cells at the injured portion, which may be a crucial manner for monocytes to emigrate from atherosclerotic plaques without rendering them into macrophages or foam cells^139^.

D-[113-122]Apo J derived from native Apo J may bind to hydrophobic domains on lipoproteins surface to avoid interactions between different LDL particles and reduce their retention, as well as lessen lipoperoxides content in lipoproteins and further sequester lipoperoxides to prevent the oxidation of LDL^69^,^140^. Similar to Apo E and Apo J mimetics, various synthetic Apo AI peptides containing 4F (L-4F and D-4F), 5A, 6F and ELK-2A2K2E have been designed and certified to be functionally analogous to native Apo AI. 4F interacts strongly with lipids on the surface of LDL and blocks lipid distribution, consequently regulating Apo B100 conformation and suppressing SMase-induced LDL aggregation^22^. D-4F exerts crucial antioxidative roles in ECs via scavenging ROS and restraining ROS production, which suppresses the activation of NADPH oxidase and the production of ROS via AMP-activated protein kinase (AMPK)/PKC pathway, alleviating oxidative injuries in ECs^141^. D-4F also promotes ECs repair and ameliorates the oxidative damage of ECs triggered by ox-LDL via Akt/AMPK/eNOS/HO-1 signaling pathway^142^. *In vitro* experiments, D-4F suppresses SMCs proliferation and migration induced by ox-LDL in dose-dependent mode. The expression of HO‐1 in SMCs is stimulated by D‐4F and the PI3K/Akt/AMPK pathway is involved in these processes. As well, this pathway mediated by D‐4F alleviates oxidative stress in SMCs via its interaction with ABCA1^143^. Moreover, 4F activates small G protein and its downstream janus kinase-2 (JAK2)/signal transducers and activators-3 (STAT3) signaling pathway through ABCA1, acting vital roles in macrophage activation^144^. Activation of STAT3 incites tristetraprolin (TTP) expression that regulates ribonucleic acid stability in macrophages^145^. Oral D-4F reduces atherosclerosis in Apo E-deficient and LDLR-deficient mice, significantly increasing cholesterol emigration from mouse macrophages by mediating cellular signal transduction^146^. D-4F may trigger intracellular cAMP release and subsequent PKA-mediated ABCA1 phosphorylation, further improving the interaction with ABCA1 and accelerating cholesterol efflux to form HDL-like particles^147^. ELK-2A2K2E, another Apo AI mimetic peptide, is reported to restrain foam cells formation through interfering VCAM-1 expression in ECs as well as monocytes activation and infiltration^148^, which stimulates messenger RNA degradation of TNF-α, MCP-1 and IL-6, and decreases their generation by modulating ABCA1/JAK2/STAT3/TTP signaling pathway^144^. Beyond that, 5A peptide also exhibits anti-atherogenic effects similar to Apo AI, and antioxidative and anti-inflammatory properties of 5A peptide are mediated mainly by ABCA1 and NF-κB signaling pathways, decreasing ROS production, ECs adhesion cytokines expression and inflammatory cells infiltration, thereby reducing the formation of foam cells^149^. 5A peptide induces macrophages cholesterol efflux via ABCA1-mediated mechanism both *in vivo* and vitro, and reduces atherosclerosis in Apo E-absent mice^150^.

## 8. Conclusion and outlook

Atherosclerosis is the underlying cause of most cardiovascular diseases and is highly associated with coronary artery disease (CAD), stroke and peripheral artery disease, resulting in high morbidity and mortality worldwide. In this review, we mainly pay attention to the biological characteristics of Apos, the roles of Apos in the pathogenesis of early atherosclerosis, and Apos as the meaningful targeting mass in the treatment of early atherosclerosis. It indicates that Apos serve as critical communicators of lipoproteins for the pro-atherogenic and anti-atherogenic processes of early atherosclerosis through influencing LDL retention and aggregation, oxidative modification of LDL, foam cells formation, and RCT in macrophage cells or foam cells. Correspondingly, Apos may be applied as the meaningful targeting mass in endogenous and/or exogenous manners for the effective treatment of early atherosclerosis.

Although the early intervention of atherosclerosis may achieve the desired therapeutic effect, long-term clinical silence may delay the optimal treatment time because atherosclerosis is a chronic progressive disease. If early treatment is not timely, acute myocardial infarction, ischemic stroke, pulmonary embolism and other severe complication may occur^151^. Therefore, from a clinical perspective, early detection and diagnosis of atherogenic risk factors are significantly important, which enable to timely intervene before the deterioration and emergence of clinical symptoms. Since increased LDL cholesterol level is a typical hazardous factor for CAD, LDL has traditionally been used for the assessment of risk associated with atherosclerosis and relevant coronary artery disease. While LDL is not a perfect predictive factor because numerous individuals with cholesterol levels within the health-associated reference range also develop atherosclersis^152^. This is partly attributed to the focusing only on LDL cholesterol and ignoring other important aspects of Apos as communicators of lipoproteins involved in lipid metabolism and atherosclerotic development. Remarkably, if Apos contribute to certain aspects of the pathogenesis of early atherosclerosis, targeting Apos-related indicators could become another potential diagnostic clue for early prevention. Pieces of evidence indicate that Apos measurements as potential communicators of lipoproteins may be better predictors and diagnostic indicators in clinical practice than traditional lipid parameters^153^. In general, higher concentrations of Apo B, Apo CII and Apo CIII, lower concentrations of Apo AI and Apo E, as well a high Apo B/Apo AI ratio, are identified as independent risk factors for CAD^45^,^126^,^154^. Apo B is superior to non-HDL-cholesterol and LDL-cholesterol as a predictor of cardiovascular risk. Introducing Apo B measurement into routine care could prevent more events than diagnosis and treatment based on non-HDL-cholesterol and LDL-cholesterol levels^155^,^156^. Apo B serves as the main transporter of atherogenic lipoproteins, and Apo AI as an anti-atherogenic agent is responsible for the reverse transport of cholesterol. The Apo B/Apo AI ratio reveals the balance between opposite forces of pro-atherosclerosis and anti-atherosclerosis. The higher the Apo B/Apo AI ratio, the more cholesterol is accumulated within the subendothelial space, hence triggering atherosclerotic development and increasing the risk of CAD^157^. Additionally, epidemiological studies have indicated that plasma Apo CIII levels alone and as a component of lipoproteins are strong and independent predictors of CAD in prospective human cohorts adjusted for classic lipid risk factors^126^,^158^. HDL or LDL-containing Apo CIII predicts a higher risk of CAD, and the negative correlation between Apo E in HDL and CAD risk was wholly covered up in Apo CIII-containing HDL^58^,^158^. On the contrary, LDL or HDL without Apo CIII could not forecast the occurrence and risk of CAD^159^. In conclusion, Apos exert a significant role in the diagnosis of atherosclerosis, and further research in this aspect may be a very prospective direction for atherosclerotic therapy in the future.

## Figures and Tables

**Table 1:** Apos exist in CM, VLDL, IDL, LDL and HDL.

Apos/lipoproteins	CM	VLDL	IDL	LDL	HDL	Reference
Apo AI	+	+	-	-	+	43, 44
Apo AII	-	-	-	-	+	43, 44
Apo AIV	+	-	-	-	+	7, 45
Apo AV	+	+	-	-	+	33, 46
Apo B48	+	-	-	-	-	37
Apo B100	-	+	+	+	-	37
Apo CI	+	+	+	+	+	47, 48, 49
Apo CII	+	+	-	-	+	47, 48, 49
Apo CIII	+	+	+	+	+	47, 48, 49
Apo E	+	+	-	-	+	33
Apo M	+	+	-	+	+	41
Apo J	-	+	-	+	+	50, 51

**Table 2:** Apos as meaningful targeting mass for treatment of early atherosclerosis.

Two available strategies	Intervening measure	Mechanisms for treatment early atherosclerosis	Reference
Endogenous Apos as meaningful targeting mass	Anti-Apo B ASO	Lessening hepatic Apo B synthesis; Lowering aortic LDL permeability; Reducing LDL retention and aggregation	122
	Mutating Apo B	Lessening LDL retention	123
	Anti-Apo CIII ASO	Lessening LDL retention	124, 125
	A specific antibody against Apo CIII	Decreasing the expression of ICAM-1 and VCAM-1 in ECs through inactivating PKCβ and NF-κB; Inhibiting monocyte recruitment	49, 124
	Statin administration for Apo CIII	Inhibiting Apo CIII-induced ECs activation and monocyte infiltration by restraining the NF-κB pathway	126
	4010B-30 for Apo AI	Regulating Apo AI gene expression partly through the peroxisome proliferators-activated receptor γ-mediated pathway; Stimulating RCT	127
	Myriocin for Apo AI	Increasing hepatic Apo AI synthesis by inhibiting of extracellular-signal-related kinase phosphorylation; Promoting RCT	128, 129
	RVX-208 for Apo AI	Upregulating Apo AI by inhibiting the bromodomain and extraterminal protein; Promoting RCT	130, 131
	Niacin for Apo AI	Preventing hepatic Apo AI clearance; Augmenting RCT	132
	Niacin for Apo M	Upregulating Apo M gene and protein expression via LXRα regulation	132
	Simvastatin for Apo M	Inducing Apo M expression through inhibiting LXRα and expediting hepatocyte nuclear factor-1α; Facilitating RCT	134
Exogenous Apos mimetic peptides as specific carriers or therapeutic agents	Ac-hE18A-NH2	Increasing PON-1 activity related to HDL; Decreasing plasma ROS and lipid hydroperoxide levels; Restraining the expression of IL-6, VCAM-1, and MCP-1 induced by lipopolysaccharide in ECs and macrophages; Motivating RCT	136, 137
	ATI-5261	Prompting RCT	136
	CS-6253	Prompting RCT	136
	Ep1.B	Reducing inflammatory monocytes infiltration; Inducing monocytes to differentiate into dendritic cells at the injured portion	139
	D-[113-122]Apo J	Reducing LDL retention; Lessening lipoperoxides content in lipoproteins and further sequestering lipoperoxides to prevent the oxidation of LDL	69, 140
	4F	Regulating Apo B100 conformation and suppressing SMase-induced LDL aggregation	22
	D-4F	Exerting antioxidative roles via AMP-activated protein kinase (AMPK)/PKC pathway; Suppressing VSMCs proliferation and migration; Augmenting RCT	141, 143, 146, 147
	ELK-2A2K2E	Interfering VCAM-1 expression in ECs as well as monocytes activation and infiltration	148
	5A	Decreasing ROS production; Reducing ECs adhesion cytokines expression and inflammatory cells infiltration; Inducing RCT	149, 150

## Abbreviations

Apos: amphiphilic apolipoproteins
LDL: low-density lipoprotein
RCT: reverse cholesterol transport
TG: triglycerides
PL: phospholipids
CM: chylomicron
VLDL: very low-density lipoprotein
IDL: intermediate-density lipoprotein
ECM: extracellular matrix
PGs: proteoglycans
ECs: endothelial cells
ICAM-1: intercellular adhesion molecule-1
VCAM-1: vascular cell adhesion molecule-1
SMCs: smooth muscle cells
ox-LDL: oxidized LDL
AαHs: amphiphilic α-helices
AβS: amphipathic β-sheet
LCAT: lecithin cholesterol acyltransferase
FC: free cholesterol
CE: cholesterol esters
LPL: lipoprotein lipase
TRL: triglyceride-rich lipoproteins
HL: hepatic lipase
CETP: cholesteryl ester transfer protein
LDLR: LDL receptor
LRP: LDL receptor-related protein
HSPG: heparan sulfate proteoglycans
GAGs: glycosaminoglycans
SMase: sphingomyelinases
PLA2: phospholipaseA2
α-CT: α-chymotrypsin
ROS: reactive oxygen species
mPGES-1: microsomal prostaglandin E synthase-1
PGE2: prostaglandin E2
PI3k: phosphatidylinositol 3-kinase
MAPK: mitogen-activated protein kinase
NADPH: nicotinamide adenine dinucleotide phosphate
GSK-3β: glycogen synthase kinase-3β
CaMKII: calmodulin-dependent protein kinase II
Nrf2: nuclear factor erythrocyte 2‐related factor 2
HO‐1: heme oxygenase‐1
DHCR24: 3β-hydroxysteroid-Δ24 reductase
PDZK1: PDZ-domain-containing protein 1
PKCα: protein kinase C alpha
β1: beta1
PTX: pertussis toxin
PC-PLC: phosphatidylcholine-specific phospholipase C
Apo ER2: apo E receptor 2
eNOS: endothelial nitric oxide synthase
NO: nitric oxide
S1P: sphingosine-1-phosphate
S1PR: S1P receptors
TNF-α: tumor necrosis factor-α
MMP-9: matrix metalloproteinase-9
RB: retinoblastoma protein
PDGF: platelet-derived growth factor
COX-2: cyclooxygenase-2
PGI2: prostacyclin
cAMP: cyclic adenosine monophosphate
CRE: cAMP-response element
ABCA1: adenosine triphosphate-binding cassette transporter A1
ASO: antisense oligonucleotide
LXRα: liver x receptor α
AMPK: AMP-activated protein kinase
JAK2: janus kinase-2
STAT3: signal transducers and activators-3
TTP: tristetraprolin
CAD: coronary artery disease
